# Environmental, Nutritional, and Therapeutic Factors That Affect Surgical Wound Healing: A Narrative Review of Experimental Findings From the Last 5 Years

**DOI:** 10.1002/hsr2.71691

**Published:** 2026-02-09

**Authors:** Heather M. Zimmerman, Akash Liyanage, Ana Danko, Joshua D. Seid, Travis Hong, Fereydoun D. Parsa

**Affiliations:** ^1^ John A. Burns School of Medicine Honolulu HI USA; ^2^ Faculty of Medicine General Sir John Kotelawala Defense University Ratmalana Colombo Sri Lanka; ^3^ Hansen's Disease Branch Hawaiʻi State Department of Health Honolulu Hawaii USA; ^4^ Department of Medical Education The University of Mississippi School of Medicine Jackson Mississippi USA; ^5^ Hawai'i Pacific Health Honolulu HI USA; ^6^ The Queen's Medical Center Honolulu HI USA

**Keywords:** closed incisional negative pressure wound therapy, healing, iodine, nutrition, radiation‐induced skin injury, surgical wounds

## Abstract

**Background and Aims:**

Wound healing is a crucial aspect of clinical outcomes following surgical procedures. Various physiological, environmental, and lifestyle factors impact healing time and quality.

**Methods:**

This review is based on the latest experimental findings pertaining to surgical wound management and healing, based on a PubMed search of experiments from the last 5 years. Topics of interest were infection control, nutrition, radiation‐induced skin injury (RSI), and closed‐incisional negative pressure wound therapy (CiNPWT). The study mainly assesses wound healing by primary intention and focuses on easily implemented therapies.

**Results:**

403 articles were screened regarding infection prevention with 336 excluded based on title and preview of content. 67 abstracts were further reviewed which yielded 32 articles for analysis. Overall, povidone‐iodine is shown to improve the rate of wound healing and helps minimize complications such as infections or wound dehiscence while creating a sterile field due to its antimicrobial properties. It remains a standard of comparison for other anti‐infectives. Nutritional factors such as veganism may impair surgical wound healing, whereas maintaining appropriate albumin levels may be protective. Employment of nutritional screenings may improve overall outcomes post‐operatively, and oral probiotics improve soft tissue healing after incision, in at least one study. Pycnogenol® and Centellicum® may have applications to improve surgical site healing, since supplementation demonstrates improved perfusion and other favorable metrics. Management of RSI is a field of much exploration, with many topicals shown effective. Recently, hydrogels and even injection of stromal vascular factor show promising results. CiNPWT is found to improve overall surgical outcomes in diverse wounds, and sponge width is an important consideration.

**Conclusion:**

Consideration of the findings discussed, along with their potential combinations and applications, may serve to improve surgical wound healing.

## 1 Introduction

Surgical site infections (SSI) occur in 2% to 4% of inpatient procedures in the United States, and are a leading cause of readmission with up to 3% of those infected postoperatively expiring [1]. While rarer in outpatient procedures, contribution to morbidity and mortality is appreciated [1]. To improve surgical wound healing, research focuses on modifying nutritional factors, environmental conditions, and therapeutic agents. The available research on this subject is vast, with many therapies in ongoing stages of development. Thus, in this narrative review, risk factors that may contribute to poor surgical wound healing are discussed, and techniques to reduce SSI of wounds of primary intention are emphasized. Thus our research question is: what are the latest, most facile to apply experimental findings within the last 5 years on modifiable environmental agents which impact surgical wound healing?

## 2 Methods

Ethics: No IRB or informed consent was sought for this review, as it falls into the category of non‐human subjects research.

**Figure 1:**
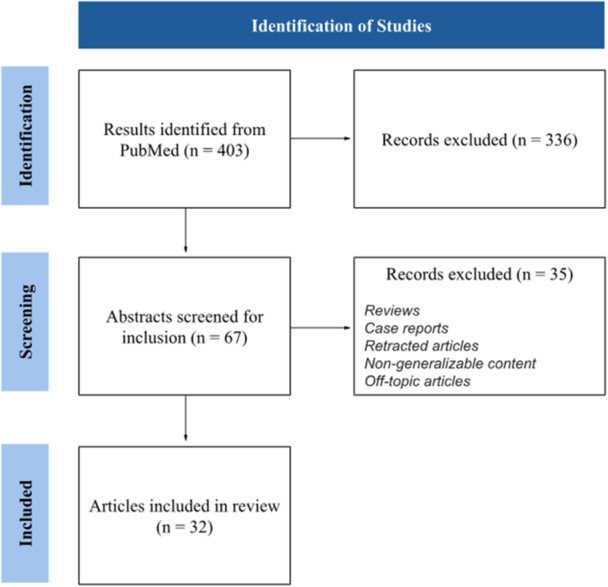
Selection process for articles included in the review, for illustrative purposes only.

For this narrative review, a list of papers was constructed, largely using a PubMed keyword search. Experimental areas of interest were collectively agreed upon and addended in the format “surgical AND wound AND healing AND [term].” Search terms included: “nutrition, iodine, closed incisional negative pressure wound therapy, and radiation‐induced skin injury. Notably, photobiomodulation and hyperbaric oxygen therapy were excluded as topics, due to the vast amount of literature which warrants another review in its entirety. Experimental techniques pertaining to wound management were of interest. Articles from within the last 5 years were screened for inclusion, while articles whose content could not reasonably be applied to cutaneous surgical wounds of primary intention, or were over 5 years old were excluded. Additional exclusions applied to case reports, series and retracted articles. Reviews were excluded unless pertaining to background information. For illustrative purposes, the overall result of our article selection process can be viewed in Figure 1. After selecting articles of interest, we collectively analyzed and extrapolated. The results of this collaborative effort is to follow.

## 3 Discussion

### 3.1 Infection Control

Infection risk is extremely high after surgery, as moisture from the environment or bodily fluids promotes bacterial growth [2]. Biofilms create a hypoxic, acidic environment, which obstructs blood flow [2]. Introduction of microbes is an inherent risk, as intraoperative wound bed contamination is common [3]. Povidone‐iodine (PI) is a versatile antimicrobial agent for infection control which is also cost‐effective and easy to apply; however, its use has fallen in and out of favor over the years, possibly due to contraindications such as skin irritation, allergy and potentially caustic nature [4, 5]. In the following section, recent studies on its effectiveness for surgical wounds and comparison with other antimicrobial agents is discussed.

One recent study detailed a specific, 16‐step “no touch protocol” that seemingly eliminated SSI in breast surgeries compared to a standard approach [5]. A retrospective review of 198 women undergoing standard procedure was compared to 167 undergoing the “no touch protocol.” This protocol included ample use of PI, glove changes, and avoidance of blunt dissection with the fingers, whereby it demonstrated a rate of infection 3.54% lower than the control with a total absence of infections (*p* = 0.017) [5]. These results are not unexpected, as a recent in vivo/in vitro study demonstrated that Methicillin‐resistant *Staphylococcus aureus* and *Pseudomonas aeruginosa* were sensitive to PI. Varying solution strengths of PI (0%–5%) were added to laboratory models after which they were cultured. In vitro, a concentration of 0.5% was required to inhibit bacterial growth, and both 0.5% and 1.25% killed all bacteria after 10 min. Although the 0.5% and 2.5% irrigations promoted pro‐growth constituents (BMP‐2, VEGF, TGF‐B1, IL‐10), 5% PI irrigation inhibited tissue healing due to increased IL‐6. During in vivo studies in rats, irrigation was not found to be thyrotoxic, nephrotoxic or hepatotoxic, at any concentration [6]. Based on this study's results, it is important to consider both the application route and strength of PI when implementing it for infection control.

The success of PI in the 16 step “no touch protocol” is not isolated, as other recent studies demonstrate its effectiveness. For example in a retrospective case‐control cohort study of 120 patients undergoing spinal fusion, the autologous bone graft was soaked in PI and wound irrigation with PI was also utilized. It was found that the PI‐soaked group had decreased overall infection rate, again 0% versus 4.03% in the non‐PI‐soaked group (*p* = 0.026). Deep infection rate was shown to be absent as well (0% vs. 3.23%, *p* = 0.047). Interestingly, a univariate logistic regression was performed to evaluate the relationship between overall infection events and different variates, revealing only BMI as significant harmful factor [7]. An additional study of hypospadias repair demonstrated that when PI was added to twice‐daily body wash within 48 h of surgery, infection rates decreased. 3 groups were compared, including body wash plus PI of 5% or 0.5%, and a retrospective body wash alone group as a control. Results varied between groups, with only the 5% povidone‐iodine group showing a significant decrease in SSI (*p* = 0.009) in comparison to control [8].

Since PI is both effective and ubiquitous it is often used as the standard of comparison or as an adjunct to other anti‐infective topicals. In a randomized controlled trial (RCT) of 102 women undergoing Cesarean Section (CS), participants were allocated into a pre‐operative PI topical group, chlorhexidine gluconate (CG) topical group, and CG shower group. Wounds were assessed at 24 h, 48 h and 7 days post‐operatively. PI alone significantly reduced erythema and edema between all 3 timepoints. However, in this study at least one SSI was attributed to the PI group and CG showering or topical use was reported to have better results for overall wound healing [9]. In another prospective randomized cohort study with differing results, 70 women were allocated into equal‐sized groups after CS and either a combination honey‐PI dressing, or PI dressing alone as control was applied. Wound sites were analyzed using the ASEPSIS score at days 5, 7, 9 and 10 post‐operative. The group utilizing a honey‐PI dressing incurred a lower mean ASEPSIS score on day 5 compared to PI dressing alone (36.14 *vs.* 37.74); however, this difference was not significant. On day 7, the experimental group still had a lower score (28.63 vs. 32.10), yet lack of significance persisted. Scores did dropsignificantly on days 9 and 10 (21.54 vs. 27.14 day 9 (*p* = 0.044); 18.26 vs. 23.86 day 10 (*p* = 0.047)). Overall, the honey‐PI group demonstrated healing 3 days quicker than control (18 vs. 21 days to heal) [10]. The efficacy and comparative capacity of PI is demonstrated yet again, in a study on rats. A linear wound incision was created in 5 rats and then treated with either PI, hypericum perforatum, tincture of benzoin, tretinoin or control. The authors found hypericum perforatum extract increased re‐epithelialization and collagen more than other topicals, and resulted in less scarring; however, tincture of benzoin and PI also demonstrated favorable results compared to control. Tretinoin was found to promote angiogenesis and collagen formation, but reduced re‐epithelialization so it was not deemed effective [11]. Based on this, further exploration into hypericum perforatum could be warranted in human studies. Lastly, although PI is often comparable to other agents for irrigation or topical application, one study reported that suture threads treated with aerosolized silver sulfadiazine may be superior at reducing microbial load compared to suture threads treated with PI or ethanol, and its use improved overall results [12].

Although SSI and other complications are often considered an inherent risk of surgery, the findings of the presented studies demonstrate that adopting validated intraoperative protocols– including the use of PI and other comparable antimicrobials– may be among the most critical factors in their prevention.

### 3.2 Dietary Factors

Nutrition is a known factor that affects surgical wound healing. Inadequate sustenance reduces immunity, skin thickness, subcutaneous fat, and collagen synthesis, all of which contribute to poor healing of all wounds [13]. According to a recent comprehensive narrative review from inception to 2023, Omega‐3 fatty acids and some amino acids enhance wound healing. Additionally, Vitamins including A, B, C and Zinc are therapeutic while Vitamin E has mixed results [14]. Expectedly, malnutrition correlated with post‐operative complications, whereas supplemental pre‐operative nutrition improves metrics [14]. The results of our review parallel these trends, with the most recent experimental studies demonstrating similar findings.

Hypoalbuminemia delays surgical wound healing, with many studies demonstrating effects on primary intention. In one case‐control study of 211 elderly inpatients undergoing hip arthroplasty, one group had normal albumin pre‐operatively, defined as > 35 g/L, whereas the other group had albumin level of < 35 g/L. Patients with hypoalbuminemia were found to have a longer LOS (14.99 ± 5.72 vs. 12.46 ± 3.16 days, *p* < 0.001) and 1.89 times higher risk for any post‐operative complication, with delayed wound healing noted at 14.3% higher in the malnourished group (OR = 0.359; 95% CI, 0.169–0.761; *p* = 0.006) [15]. Similar results were noted in an abstract of a hospital‐based, prospective study of 330 coronary artery bypass graft patients, where researchers found a statistically significant correlation between albumin, blood glucose and sternal wound healing [16]. Yet again, a retrospective review of 554 patients undergoing posterior lumbar interbody fusion surgery revealed similar results. Four groups were analyzed: Surgical wound dehiscence, normal wound healing, SSI, and non‐SSI. Pre and post‐operative hypoalbuminemia were defined as < 3.5 g/dL or < 3.0 g/dL, and then compared to wound dehiscence and SSI. Multivariate logistic regression demonstrated that pre‐operative hypoalbuminemia corresponded to wound dehiscence (*p* = 0.024, OR = 4.16, 95% CI 1.203–14.44) as did post‐operative hypoalbuminemia (*p* < 0.001, OR = 5.22, 95% CI 2.84–9.58). Pre‐operative hypoalbuminemia was also a risk factor for SSI (*p* = 0.04, OR = 5.69, 95% CI 1.08–29.88) [17]. Another retrospective review of pre‐operative nutritional status investigated albumin as a predictor of healing in 302 amputees. Researchers did not find any significant difference in post‐operative complication within 30 days of surgery (dehiscence, hematoma, or infection) at albumin level < 3.2 g/dL; however, at pre‐albumin < 10 mg/dL the rate of infection significantly increased (12.1% vs. 3.3%; *p* = 0.014), and at < 9 mg/dL both hematoma and infection significantly increased (6.0% vs. 0.7%; *p* = 0.021; 12.0% vs. 4.3%; *p* = 0.039) [18]. An additional abstract describing a study of 152 maxillofacial trauma patients found that unspecified post‐operative complications were significantly related to female gender (odds ratio=2.08, 95% CI, 1.02–4.21; *p* = 0.04) and increasing number of procedures (*p* = 0.02) versus a non‐significant relationship to nutritional metrics of albumin and hemoglobin [19]. While most studies indicate albumin level as a strong predictor of wound complications, the incongruence of at least two studies calls into question whether the albumin threshold may vary between study populations. Perhaps other indicators, such as pre‐albumin, may contribute to assessing its precise role in determining pre‐operative nutritional status and risk of complication. Furthermore, one recent study demonstrated that mice fed a low‐protein, high‐carbohydrate, and/or fat diet experienced accelerated wound healing. Healing was delayed by high‐protein or very high‐fat intake; thus, the interplay of serum albumin and macronutrient intake in surgical patients should additionally be investigated [20].

Regarding use of pre‐operative nutritional screenings to improve or predict wound healing, several recent studies exist that demonstrate its impact on wounds of both primary and secondary intention. For example, a single center case‐control cohort study of 1,031 diabetic foot episodes among 586 patients revealed that the 19% who underwent professional dietary guidance and intervention had significant reduction in median LOS by 3 days (*p* = 0.02) and shorter courses of antibiotics by 4 days (*p* = 0.010). Lastly, those patients who were deemed lower‐nutritional risk on admission had lower failure rates after surgery (Cox regression analysis; hazard ratio 0.2, 95% CI 0.1–0.7) [21]. Nutritional intervention has also proven effective in another retrospective comparative study of 60 patients undergoing gastrointestinal surgery. Patients were assigned to either an experimental or control group, with the experimental group receiving a combination of enteral and/or parenteral nutrition, whereas the control group only received conventional support. SSI in the experimental group was significantly reduced compared to control (10% vs. 30%, *p* < 0.05), and the wounds healed faster (10.35 ± 2.42 days vs. 14.42 ± 3.15 days, *p* < 0.05) [22]. Effectiveness of pre‐operative nutritional support was revealed by a RCT conducted on 103 lumbar surgery patients, 37% of whom were considered malnourished pre‐operatively (albumin < 3.5 g/dL). 46 were given supplementation (protein shake twice‐daily), whereas the remainder received standard nutrition. Wound healing complications were lower in the supplemented group (3.4% vs. 17.9%, *p* < 0.05) [23]. In another observational study of 440 general surgery patients, patients deemed nutritionally compromised pre‐operatively (defined by BMI, serum albumin and Nutritional Risk Screening Score) were found to have significantly worse outcomes compared to those who were nutritionally sound. LOS was found to be lower in the nutritionally sound group (6.53 + /−2.31 days vs. 9.97 + /1 3.58 days, *p* < 0.001) and post‐operative infections were significantly decreased (13.64% vs. 36.36%, *p* < 0.001) as was the incidence of delayed wound healing (9.09% vs. 22.73%, *p* < 0.001) [24]. The Controlling Nutritional Status (CONUT) score has also been used in a recent retrospective study of open surgery for ischemic tissue loss. 174 limbs in 147 patients were included, and those with normal to mild malnutrition (CONUT score ≤ 4) demonstrated improved wound healing compared to those with moderate‐severe malnutrition (CONUT score ≥ 5). More nourished individuals had 50% of their wounds healed 97% of the time at 2 years, versus only 79% of the time in the malnourished group (*p* < 0.001). Overall, CONUT score independently predicted healing (hazard ratio 0.63; 95% CI, 0.41–0.98; *p* = 0.038) [25].

An additional concern when analyzing surgical wound healing is intentional dietary restrictions For example, a recent prospective dermatological study showed that surgical scars may be at risk of impaired healing in vegans. 21 vegans were compared to 21 omnivores, all undergoing non‐melanoma skin cancer excision. The Scar Cosmesis Assessment and Rating (SCAR) scale was used to evaluate for complications and wound healing. Although not statistically significant, the sole SSI occurred in a vegan. Separation of the closure site only occurred in vegans (29% vs. 0% *p* = 0.008), and at 6‐months follow‐up vegans had higher median scar spread (3 (range 1–4) vs. 1 (range 1–3)), atrophy (3 (range 1–4) vs. 0 (range 0–2)) and SCAR score (9 (range 5–14) vs. 5 (range 4–9)) (*p* < 0.001); additionally, vegans had reduced iron and B12 compared to omnivores (48.71 + /−8.71 vs. 82.76 + /−24.26 ug/dl; 174.29 + /−19.05 vs. 330.62 + /−173.4 pg/mL; *p* < 0.001). This lends credence to the idea of amino acid and vitamin deficiencies impeding the healing process, as both vegetarians and vegans are known to be at risk of inadequate intake of at least iron and B12, due to a potentially imbalanced diet [14, 26].

Further advances in nutritional approaches to healing have been demonstrated in more niche studies. For example, a triple‐blind RCT on 74 primiparous women demonstrated that when women with episiotomies of < 5 cm were given daily *Lactobacillus casei* capsules from birth to 2 weeks postpartum, they experienced accelerated healing. The wound healing severity score of the probiotic group decreased when compared to placebo between all stages (discharge to 5 + /−1 days after birth, to 15 + /−1 days after birth). The adjusted mean difference was ‐0.50 between the experimental and control group (95% CI, −0.96 to −0.05, *p* = 0.03). The authors concluded that *Lactobacillus casei*, when taken orally, improves healing of episiotomies, and called for further studies on topical use [27]. Although episiotomies are mostly performed on the vaginal mucosa, investigation of probiotics' effects on cutaneous wounds seems promising for future studies. An additional abstract of a registry study demonstrated that women taking oral, supplemental Pycnogenol® and Centellicum® (PYCE) for 4 weeks had improved skin elasticity on ultrasound (*p* < 0.05) when compared to control. Importantly, both groups were nutritionally comparable at baseline and followed similar skincare regimens. Additionally, transcutaneous pO2/pCO2 measurements, hydration and blood flow were increased in the PYCE group, (*p* < 0.05). The authors concluded that PYCE should be investigated as an adjunct to improve wound healing, since skin elasticity and perfusion are critical for cutaneous wound healing [28].

Nutrition's relationship to cutaneous surgical wound healing is highly demonstrated, including in recent experimental studies on various types of operations. Hypoalbuminemia appears to be the most studied culprit for poor wound outcomes, with dietary restrictions such as veganism shown to affect long term scar appearance. For both, conflicting or absent evidence suggests further studies may be necessary to confirm these variables' precise role. Nutritional screenings are generally beneficial, with various types studied. More novel studies have explored the effects of oral probiotic supplementation on mucosal healing rates or oral supplementation with Pycnogenol®/Centellicum® and its effect on skin perfusion and elasticity. These studies hold promise for further exploration regarding their application for cutaneous surgical wounds.

### 3.3 Radiation‐Induced Skin Injury

As cancer treatments advance, radiation therapy is becoming more precise; however, healthy tissue is inevitably exposed, with up to 95% of patients experiencing radiation‐induced skin injury (RSI) during tumor radiotherapy [29]. RSI is of particular concern for surgical wounds as tumor excision and reconstructive surgery is often performed before or after radiation.

Basic, evidence‐based steps can be taken to manage RSI, including limiting the radiation field and shielding the skin [2, 29]. Metallic dressings, topicals (including biologics such as epidermal growth factor) and even natural therapies such as calendula may improve RSI [29]. Recently, a glucopeptide‐superstructured hydrogel promoted surgical wound healing following neoadjuvant radiotherapy in mice, in comparison to control of hyaluronic acid alone. The authors postulate it inhibited ROS and promoted angiogenesis. Additionally, a reduction in DNA damage and suppression of *senescence‐associated secretory phenotype* mitigated radiation‐induced cellular senescence [30]. In another study, stromal vascular factor (SVF) was harvested from patients with RSI. Its injection favorably reduced RSI in mice at 2 and 3 weeks post‐irradiation when compared to control. Notably, adipose‐derived stem cells are present at up to 10% of volume in the solution used. 5 patients were also administered their own SVF in this study, and experienced similar results. The authors acknowledge that the therapeutic component of SVF is yet to be identified [31]. Adding to this finding, adipose‐derived mesenchymal stem cells were shown to lessen RSI in guinea pigs when conjoined with low intensity ultrasound [32]. An earlier study also demonstrated that enrichment of human lipoaspirate with CD34 + CD146 + , CD34 + CD146‐, or CD34+ human adipose stem cells improved scalp RSI when injected into irradiated mice [33].

Based on these recent studies, further exploration into exact mechanisms and interplay within the wound microenvironment are duly warranted.

### 3.4 Closed Incisional Negative Pressure Wound Therapy

Negative pressure therapy (NPT) for open wounds has been utilized for at least two decades, with closed‐incisional negative pressure wound therapy (CiNPWT) becoming more mainstream [34]. In both open and closed wounds, NPT increases blood flow and oxygenation while removing exudate. CiNPWT may additionally prevent SSI through maintenance of sterility, reduction of seromas due to application of pressure, and off‐loading tension across the wound site itself [34]. The following section will explore the most recent experimental data on CiNPWT for a variety of surgical wounds.

One recent study compared a standard patch dressing to CiNPWT for oncologic excision and found that at 14 days post‐operative 30.7% of the control group experienced complications, compared to only 7.7% of the CiNPWT group. Secondarily, 15.4% of the control group had delayed wound healing versus 0% of the CiNPWT group. Additionally, LOS was 1.2 days longer in control [35]. Another retrospective, multi‐surgeon study compared CiNPWT to standard dressing for 79 mammaplasty patients. Early dehiscence was significantly decreased in the CiNPWT group at only 1 in 44 (2%) of breasts versus 16 in 144 (14%) of breasts dressed with standard dressing (*p* = 0.003). Further, the relative risk reduction was noted to be 84% and 2 patients in the standard dressing group required debridement versus 0 in the CiNPWT group [36]. In a retrospective case‐control study involving 106 of 271 anterior lateral thigh flap donor sites, CiNPWT significantly decreased both wound dehiscence and average post‐operative LOS relative to sites without CiNPWT (2.8% vs. 9.0%, *p* = 0.04; 19 ± 8 days vs. 21 ± 11 days, *p* = 0.03). Additionally, CiNPWT resulted in fewer revisions (4.7% vs. 14.0% *p* = 0.010). This same study showed slightly improved scar appearance based on responses to the Vancouver Scar Scale and Patient Observer Scar Assessment Scale (POSAS) [37]. When CiNPWT was applied to infectious scars after surgery in 25 patients, retrospective review revealed that it reduced recurrence of infection on 1‐year follow‐up. Recurrence in the prior year was 6.40 times, versus 0 times in the year following application (*p* < 0.0001). Secondarily, scar quality improved, POSAS score decreased (81.60 pre‐operatively vs. 25.36 1‐year post‐operatively, *p* < 0.0001). Importantly, infectious scars were defined as either hypertrophic or keloids and meeting all 3 criteria: infection greater than 1 month, recurrence of infection 2 or more times, localized sinus in the deep region. Although patients served as their own control in this study, the positive results are clearly demonstrated [38]. Lastly, a retrospective study compared 100 infrainguinal incisions that applied CiNPWT versus 138 that did not. SSI occurred in only 5 utilizing CiNPWT versus 13 without (*p* = < 0.05). Fewer seromas were noted in the CiNPWT group (22 vs. 28), but the difference was not significant. Importantly, the authors noted that a standardized scale was used to rate SSIs (American College of Surgeons National Surgical Quality Improvement Plan SSI Criteria) whereas seroma was diagnosed clinically based on imaging (Computed Tomography, Ultrasound) and fine needle aspiration [39]. This draws into question the exact parameters which define these outcomes, and whether subjectivity of observations may introduce bias into results.

Newer, more specialized experimental applications of CiNPWT include spinal surgeries, with a 2‐year prospective observational study noting that when compared to standard dressing, CiNPWT significantly reduced SSI in 118 of 274 patients requiring instrumentation on 60 day follow‐up (3.2 vs. 11.4%, *p* = 0.03). Notably, if decompression was used alone the difference was not statistically significant (4.2 vs. 9.1%, *p* = 0.63) [40]. Results of this study expose the question of whether application of CiNPWT to all wound and operation types is essential. Regarding qualitative experiences with novel wounds, a specialized CiNPWT system has also been evaluated for colo‐rectal surgery sites. One prospective study of 30 patients noted that the majority of patients (75.9%) felt it was very comfortable for use. It also resulted in 0 SSIs [41]. Additionally, in practice, sponge width may affect the outcome, with one laboratory model demonstrating that wider sponges may increase risk of dehiscence. 3 sponge widths were compared and it was found that a 3 cm sponge required greatest force of dehiscence at 43.0 N vs. 38.7 for 6 cm and 36.4 for 9 cm (*p* < 0.001) [34].

CiNPWT demonstrates effectiveness at reducing surgical site complications. Important considerations are the utility of its application to all wound types, along with potential for bias in outcome measures which rely on clinical or patient observations. Further, exact sponge width may play a role in the reduction of improvements, but further in vivo studies are needed.

## 4 Conclusion

Novel therapies and unique situations have been reviewed pertaining to surgical wound healing. Some studies support further investigation, such as effective management of RSI. We found that vegans and other malnourished individuals may require special attention due to the potential for an unbalanced diet. Lastly, microbial burden and poor perfusion are indubitably related to delayed surgical wound healing, and PI or CiWNPT has proven effective in many cases.

## Author Contributions

**Heather M Zimmerman:** formal analysis, investigation, validation, writing – original draft. **Akash Liyanage:** formal analysis, validation, writing – review and editing. **Ana Danko:** formal analysis, software, writing – review and editing. **Joshua D. Seid:** formal analysis, writing – review and editing. **Travis Hong:** formal analysis, writing – review and editing. **Fereydoun D. Parsa:** conceptualization, formal analysis, supervision, visualization, writing – review and editing.

## Funding

The authors received no specific funding for this work.

## Conflicts of Interest

The authors declare no conflicts of interest.

## Transparency Statement

The lead author Heather M. Zimmerman affirms that this manuscript is an honest, accurate, and transparent account of the study being reported; that no important aspects of the study have been omitted; and that any discrepancies from the study as planned (and, if relevant, registered) have been explained.

## Data Availability

The authors confirm that the data supporting the findings of this study are available within the article. Further records are available upon request and stored in an electronic repository.
